# Defining Optimal Doses of Liposomal Amphotericin B Against *Candida auris:* Data From an In Vitro Pharmacokinetic/Pharmacodynamic Model

**DOI:** 10.1093/infdis/jiad583

**Published:** 2023-12-18

**Authors:** Maria-Ioanna Beredaki, Ioannis Sanidopoulos, Spyros Pournaras, Joseph Meletiadis

**Affiliations:** Clinical Microbiology Laboratory, Attikon University Hospital, Medical School, National and Kapodistrian University of Athens, Athens, Greece; Clinical Microbiology Laboratory, Attikon University Hospital, Medical School, National and Kapodistrian University of Athens, Athens, Greece; Clinical Microbiology Laboratory, Attikon University Hospital, Medical School, National and Kapodistrian University of Athens, Athens, Greece; Clinical Microbiology Laboratory, Attikon University Hospital, Medical School, National and Kapodistrian University of Athens, Athens, Greece

**Keywords:** liposomal amphotericin B, *Candida auris*, pharmacokinetics/pharmacodynamics, optimal dose

## Abstract

**Background:**

*Candida auris* isolates exhibit elevated amphotericin B (AMB) minimum inhibitory concentrations (MICs). As liposomal AMB (L-AMB) can be safely administered at high doses, we explored L-AMB pharmacodynamics against *C. auris* isolates in an in vitro pharmacokinetic/pharmacodynamic (PK/PD) dilution model.

**Methods:**

Four *C. auris* isolates with Clinical and Laboratory Standards Institute (CLSI) AMB MICs = 0.5–2 mg/L were tested in an in vitro PK/PD model simulating L-AMB pharmacokinetics. The in vitro model was validated using a *Candida albicans* isolate tested in animals. The peak concentration (C_max_)/MIC versus log_10_ colony-forming units (CFU)/mL reduction from the initial inoculum was analyzed with the sigmoidal model with variable slope (E_max_ model). Monte Carlo analysis was performed for the standard (3 mg/kg) and higher (5 mg/kg) L-AMB doses.

**Results:**

The in vitro PK/PD relationship C_max_/MIC versus log_10_ CFU/mL reduction followed a sigmoidal pattern (R^2^ = 0.91 for *C. albicans*, R^2^ = 0.86 for *C. auris*). The C_max_/MIC associated with stasis was 2.1 for *C. albicans* and 9 for *C. auris*. The probability of target attainment was >95% with 3 mg/kg for wild-type *C. albicans* isolates with MIC ≤2 mg/L and *C. auris* isolates with MIC ≤1 mg/L whereas 5 mg/kg L-AMB is needed for *C. auris* isolates with MIC 2 mg/L.

**Conclusions:**

L-AMB was 4-fold less active against *C. auris* than *C. albicans. Candida auris* isolates with CLSI MIC 2 mg/L would require a higher L-AMB dose.

## Teaser

A validated in vitro PK/PD dilution model showed that liposomal amphotericin B (L-AMB) was 4-fold less active against *Candida auris* than *Candida albicans.* While standard dose is sufficient for wild-type *C albicans*, *C auris* isolates would require a higher L-AMB dose.

*Candida auris* has rapidly emerged as a global public health challenge and a multidrug-resistant (MDR) pathogen, since its first isolation in 2009, causing concern in the medical community worldwide. While resistance to antifungals is rather limited in other *Candida* spp, resistance in *C. auris* is more common [1, 2]. Indeed, based on published proposed epidemiological cutoff value (ECV) [3], *C. auris* isolates can demonstrate elevated minimal inhibitory concentrations for azoles, polyenes and echinocandins [3], even though careful interpretation is required for the susceptibility categorization of the isolates, due to the lack of established clinical breakpoints.

*Candida auris* isolates are usually resistant to fluconazole and therefore echinocandins are considered the first-line treatment for those infections. As emergence of resistance to echinocandins has been described [4], amphotericin B (AMB) remains the only active drug for MDR isolates, although resistance rates 0–30% with different methodologies have been reported [5–7]. Nevertheless, AMB has been described as the only in vitro fungicidal agent against *C. auris*, unlike echinocandins [8]. This, alongside the fact that AMB is the only alternative treatment to echinocandins for *C. auris* infections, highlights the need for in-depth study of AMB's activity.

Liposomal amphotericin B (L-AMB) can be safely administered at higher doses, thus allowing greater drug exposures with lower risk of toxicity. However the optimal dose of L-AMB for *C. auris* infections has not been defined. To date, there are no pharmacokinetic/pharmacodynamic (PK/PD) data on L-AMB activity against *C. auris* isolates that would help to describe the PK/PD relationship, define a clinically relevant PK/PD target and its magnitude, and determine optimal dose of L-AMB against *C. auris* infections. In addition, the Centers for Disease Control and Prevention (CDC) tentative breakpoint for resistance [9] overlaps with the proposed ECV [3] of 2 mg/L, questioning the effectiveness of standard dose of 3 mg/kg L-AMB against *C. auris* infections. Therefore, in the present study, we investigated L-AMB pharmacodynamics against *C. auris* isolates with different minimum inhibitory concentrations (MICs) simulating LAMB pharmacokinetics in humans using an in vitro PK/PD dilution model, validated based on animal data of experimental candidiasis.

## METHODS

### Isolates

Four *C. auris* isolates, with Clinical and Laboratory Standards Institute (CLSI) AMB MIC 0.5–2 mg/L were tested (kindly provided by J. Meis, Canisius Wilhelmina Hospital, Nijmegen, The Netherlands) (Table 1). The in vitro model was validated using one *C. albicans* K1 isolate (kindly provided by David Andes, University of Wisconsin), previously tested in an animal model of disseminated candidiasis [10] and one well-characterized AMB-resistant *C. albicans* isolate SSI-2699 [11] (kindly provided by Maiken C. Arendrup, University of Copenhagen, Denmark). Broth microdilution testing for AMB and L-AMB was performed in triplicate, in accordance with CLSI M27-A3 [12], using standard RPMI 1640 medium (0.2% dextrose).

**Table 1.** In Vitro Susceptibility of *Candida albicans* and *Candida auris* Isolates Tested in the Present Study

*Candida* Isolates	Resistance Mechanism/Clade	MIC, mg/L, Median (Range)	
		Amphotericin B Deoxycholate	Liposomal Amphotericin B
*C. albicans* K1	WT	0.25 (0.25)	0.25 (0.25)
*C. albicans* SSI-2699	*fks*1 (S649P), *Erg*2 (F105fs)	>16	16
*C. auris* 51	Clade I	1 (1)	1 (1)
*C. auris* 52	Clade I	2 (1–2)	4
*C. auris* 55	Clade I	0.5 (0.5)	0.5 (0.5)
*C. auris* 60	Clade II	0.5 (0.5)	0.125 (0.06–0.125)

Abbreviations: MIC, minimum inhibitory concentration; WT, wild type.

For the in vitro PK/PD studies, the isolates were stored in sterile normal saline with 10% glycerol at −70°C and revived by subculturing on Sabouraud dextrose agar (SDA) plates supplemented with gentamicin and chloramphenicol (SGC2, bioMérieux) to ensure purity and viability. Inoculum suspension of the subcultured yeasts was prepared in sterile normal saline and adjusted to a final inoculum of 10^4^ colony-forming units (CFU)/mL. The CFU number was confirmed by quantitative cultures on SDA plates.

### Antifungal Drugs and Medium

AMB (Sigma-Aldrich, Athens, Greece) was supplied as pure powder. Stock solutions of 5 mg/mL were prepared in sterile dimethyl sulfoxide (CarloErbaReactifs-SDS, Val de Reuil, France) and stored at −70°C until use. L-AMB (AmBisome, Gilead Sciences) was reconstituted according to the manufacturer's instructions to a final concentration of 4 mg/mL. RPMI 1640 medium with l-glutamine, without bicarbonate (Sigma-Aldrich, Athens, Greece), buffered to pH 7.0 with 0.165 M morpholinepropanesulfonic acid and supplemented with 100 mg/L chloramphenicol (AppliChem GmbH, Darmstadt, Germany), was used as the growth medium in the in vitro PK/PD studies.

### In Vitro PK/PD Model

A previously validated one-compartment PK/PD dilution model simulating in vivo pharmacokinetics was used [13, 14]. In brief, the model consists of a 250-mL culture vessel (conical glass flask) (internal compartment [IC]) containing fresh RPMI-1640 medium to an initial volume of 5 mL, for each *C. auris* isolate and L-AMB dosing regimen simulated. The culture vessel is connected to a peristaltic pump (Minipuls Evolution, Gilson Inc), adding fresh medium in order to dilute its content at a rate as the clearance of L-AMB in human plasma. The IC was covered with aluminum foil to minimize light exposure and placed on a heated (37°C) magnetic stirrer, while its volume increased over time reaching approximately 100 mL at 48 hours. Previous studies using the same model indicated that 48 hours is sufficient time to describe the pharmacodynamics, since maximum growth in drug-free control is achieved after 24 hours, regrowth does not occur in drug-containing flasks after 24 hours, and PK/PD indices did not differ significantly between 48 and 72 hours [14, 15].

### Pharmacokinetic Analysis

Different L-AMB drug exposures with peak concentrations (C_max_) 0.25–64 mg/L and an average half-life of 9 hours were simulated in the in vitro model. Drug concentrations were added at the corresponding C_max_ values in the in vitro model once daily for 48 hours. Higher and lower than the clinically achievable L-AMB exposures were evaluated in order to better describe the exposure–effect relationship. Drug levels were determined using a microbiological diffusion assay using a *Paecilomyces variotii* strain as previously described for AMB deoxycholate [16]. No antifungal activity of liposomes without AMB was found. Due to nonlinearity between inhibition zones and L-AMB concentrations >1 mg/L, samples from the IC with expected values >1 mg/L were first diluted so that the concentration would fall in the linearity range of the bioassay. The lowest limit of detection was 0.03 mg/L for the partial growth inhibition zone (80%). Interexperimental variability was assessed in replicate experiments.

### Pharmacodynamic Analysis

To estimate the fungal load inside the IC of each L-AMB dosing regimen, 500-μL samples were collected at regular intervals up to 48 hours and 10-fold serially diluted in normal saline, and 20 μL was subcultured on Sabouraud dextrose agar plates. Plates were incubated at 30°C for 24 hours and colonies were counted at each dilution. Dilutions that yielded 10–50 colonies were used to determine the log_10_ CFU/mL at each timepoint. Time-kill curves were constructed by plotting log_10_ CFU/mL over time. No carryover effect was found in preliminary experiments at L-AMB concentrations ≥16 times the MIC by comparing CFU/mL after spotting, spreading, or washing after centrifuging the samples.

### Validation of the In Vitro PK/PD Model

The in vitro PK/PD model was validated using previously published in vivo results in a neutropenic model of disseminated candidiasis in mice, which were infected with the same *C. albicans* K1 strain used in the present study, and treated intraperitoneally with increasing L-AMB doses 0.312–80 mg/kg once daily for 72 hours [10]. In addition, an AMB-resistant *C. albicans* was also tested. L-AMB exposures in mice with C_max_ 0.125–128 mg/L and average half-life of 11 hours were simulated in the in vitro model. The log_10_ CFU/mL and drug levels were determined at regular intervals. L-AMB exposure (change in log_10_ CFU/mL from t = 0 hours vs C_max_/MIC) after 48 hours of incubation, associated with a fungistatic effect (no log_10_ CFU/mL reduction compared to the initial inoculum), was calculated with nonlinear regression analysis using the sigmoidal model with variable slope (E_max_ model) and compared with the in vivo PK/PD target associated with a fungistatic effect after 3 days of treatment (change in log_10_ CFU/kidneys vs C_max_/MIC), as described below. Two independent experiments were conducted.

### PK/PD Analysis

The PK/PD index C_max_/MIC ratio was calculated for each simulated dose, isolate, and experiment. The drug exposure–response relationship, expressed with the 48 hours log_10_ CFU/mL reduction at each dosing regimen and isolate compared to the start of therapy values versus C_max_/MIC, was analyzed with the E_max_ model described by the equation E = E_max_*EI^n^/(EI_50_^n^ + EI^n^) where E is the growth rate (dependent variable), E_max_ is the maximum growth rate, EI is the exposure index C_max_/MIC, EI_50_ is the exposure index C_max_/MIC corresponding to 50% of E_max_, and n is the slope of the exposure-effect relationship (Hill coefficient). Because the volume of the in vitro PK/PD model increased over time, a similar analysis was performed using the actual log_10_ CFU after multiplying the CFU/mL with the volume at 48 hours. All data were analyzed using the statistics software package GraphPad Prism version 5.0 for Windows (GraphPad Software, San Diego, California). All experiments were repeated twice.

### Prediction of PK/PD Target Attainment

In order to bridge the in vitro data with clinical outcome, Monte Carlo simulation analysis was performed for 5000 patients receiving the standard (3 mg/kg q24h intravenously [i.v.]), as well as the higher (5 mg/kg q24h i.v.) dose, achieving blood levels corresponding to a mean ± standard deviation L-AMB C_max_ 21.87 ± 12.47 mg/L and 83 ± 35.2 mg/L [17], respectively. The percentage of patients attaining the in vitro C_max_/MIC corresponding to a fungistatic effect of L-AMB compared to the initial inoculum (this was found to correlate with clinical breakpoints of *C. albicans* as shown below) was calculated for *C. albicans* or *C. auris* isolates with CLSI MICs 0.25–8 mg/L. Recently published MIC distribution data for *C. albicans* [18] and *C. auris* [3] with CLSI were used.

## RESULTS

### Isolates

Table 1 summarizes the AMB/L-AMB in vitro susceptibilities against the *C. albicans* and *C. auris* isolates tested. MIC values of AMB and L-AMB were the same for most isolates except 2 isolates for which L-AMB MICs were one 2-fold dilution higher and two 2-fold dilutions lower than AMB MICs, respectively. More specifically, AMB MICs were 0.25–2 mg/L, for all isolates, while L-AMB MICs were 0.125–4 mg/L.

### Validation of the In Vitro PK/PD Model With *C. albicans*

#### Pharmacokinetics

L-AMB doses were well simulated in the in vitro model. The calculated C_max_ in the IC was within 20% of the target C_max_ of 0.125–128 mg/L with mean half-life (t_1/2_) of 8 (range, 7–11) hours.

#### Pharmacodynamics

**Figure 1.**
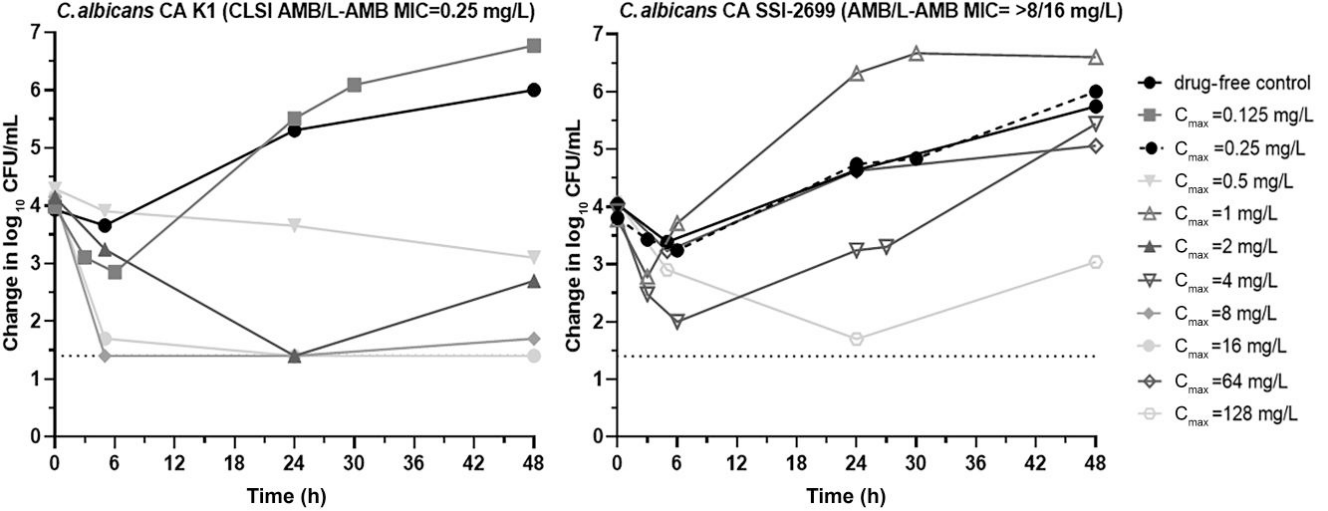
Time-kill curves in the in vitro pharmacokinetic/pharmacodynamic model simulating animal q24h liposomal amphotericin B dosing regimen against *Candida albicans* isolates targeting different maximum concentration values with a half-life of 8 (range, 7–11) hours. Abbreviations: AMB, amphotericin B; CFU, colony-forming units; CLSI, Clinical and Laboratory Standards Institute; C_max_, peak concentration; L-AMB, liposomal amphotericin B; MIC, minimum inhibitory concentration.

**Figure 2.**
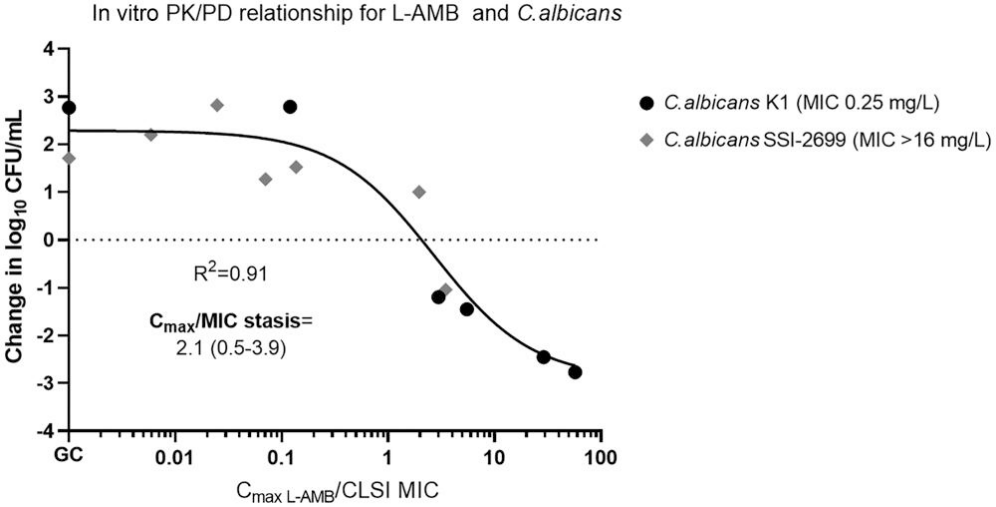
In vitro pharmacokinetic/pharmacodynamic (PK/PD) relationship of liposomal amphotericin B for the *Candida albicans* isolates tested in the in vitro PK/PD model using the 48-hour change in log_10_ colony-forming units/mL vs peak concentration/minimum inhibitory concentration compared to the initial inoculum. Abbreviations: CFU, colony-forming units; CLSI, Clinical and Laboratory Standards Institute; C_max_, peak concentration; GC, drug free growth control; L-AMB, liposomal amphotericin B; MIC, minimum inhibitory concentration; PK/PD, pharmacokinetic/pharmacodynamic.

A reduction (>1 log_10_ CFU/mL) in the fungal load of *C. albicans* K1 compared to the initial inoculum was observed at 48 hours for the highest L-AMB doses with C_max_ ≥8 mg/L, while for the lowest doses, regrowth was observed as early as 6 hours of treatment, in agreement with in vivo data [10]. No killing was observed for the AMB-resistant isolate *C. albicans* SSI-2699 at any L-AMB exposure except for C_max_ 128 mg/L (Figure 1). The in vitro C_max_/MIC_48h_ log_10_ CFU/mL relationship followed a sigmoid curve (R^2^ = 0.91) with a mean C_max_/MIC associated with a fungistatic effect of 2.1 (Figure 2), very similar to that required for stasis in mice kidneys (1.6–3.8 C_max_/MIC), based on L-AMB concentration at the site of infection [10].

#### Monte Carlo Simulation

The probability of target attainment (PTA) for the in vitro static PK/PD target with the standard dose of 3 mg/kg of L-AMB was >95% for *C. albicans* isolates with CLSI AMB MICs ≤2 mg/L, thus covering the entire wild-type (WT) *C. albicans* population (ECV 2 mg/L), further validating the in vitro model, since standard L-AMB 3 mg/kg q24h i.v. has proven clinical activity against *C. albicans* infections.

### In Vitro PK/PD Studies for *C. auris*

#### Pharmacokinetics

**Figure 3.**
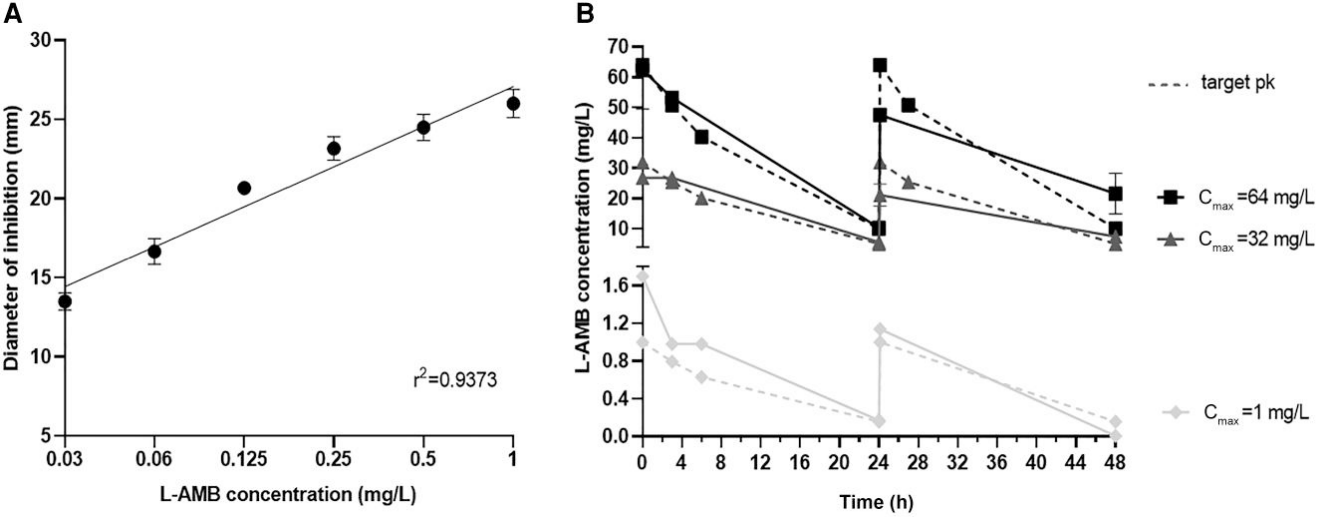
*A*, Standard curve of the diameter of inhibition zone (y-axis) – liposomal amphotericin B (L-AMB) concentration (x-axis) showed acceptable linearity in the range of 0.03–1 mg/L. *B*, Representative time-concentration profile of simulated q24h L-AMB dosing regimens in the in vitro pharmacokinetic/pharmacodynamic model for *Candida auris* isolates. Data represent drug levels in the internal compartment of the in vitro model (solid lines) and the respective target values (broken lines). Error bars represent standard errors. Abbreviations: C_max_, peak concentration; L-AMB, liposomal amphotericin B.

The time-concentration profile of the monotherapy against *C. auris* isolates is shown in Figure 3. The pharmacokinetic parameters of L-AMB were well simulated in the in vitro model with an average half-life of 10 (range, 5–12) hours and with L-AMB C_max_ concentrations within 10% of target values. As human L-AMB PK is biphasic within the 24-hour dosing period, the latter half-life corresponds to the second longer distribution half-life, which covers most of the 24-hour dosing period. As C_max_ is the PK/PD driver for L-AMB, the absence of the initial shorter elimination phase will not affect L-AMB PD.

#### Pharmacodynamics

**Figure 4.**
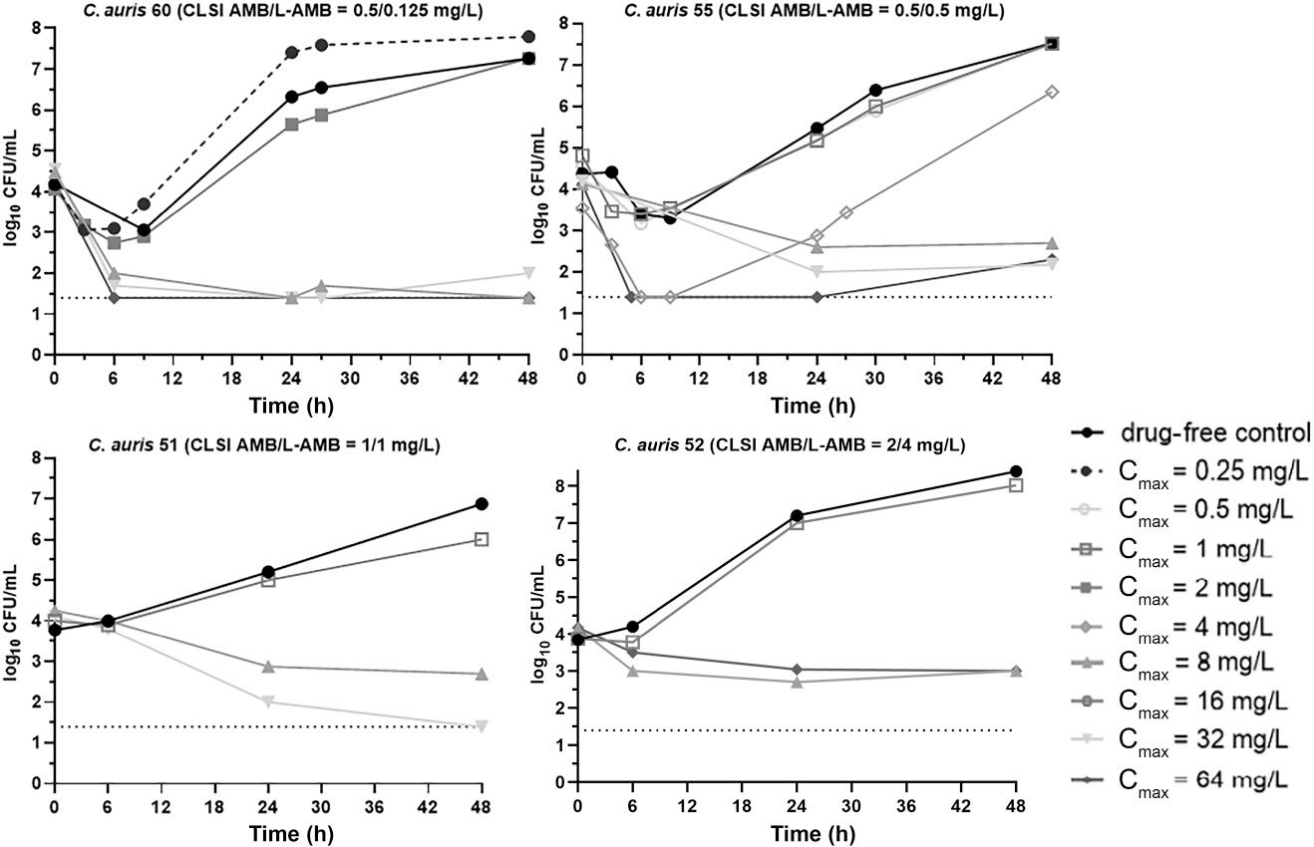
Time-kill curves in the in vitro pharmacokinetic/pharmacodynamic model for each simulated liposomal amphotericin B (L-AMB) dosing regimen against the 4 *Candida auris* isolates with increasing amphotericin B/L-AMB minimum inhibitory concentrations. Abbreviations: AMB, amphotericin B; CFU, colony-forming units; CLSI, Clinical and Laboratory Standards Institute; C_max_, peak concentration; L-AMB, liposomal amphotericin B.

**Figure 5.**
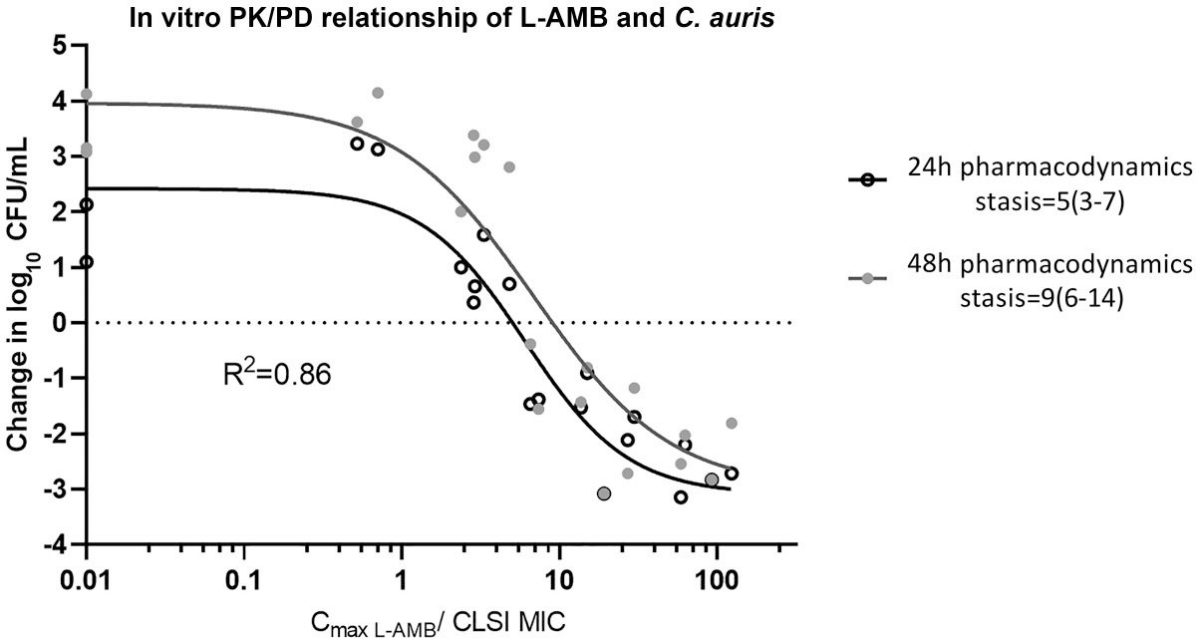
In vitro pharmacokinetic/pharmacodynamic relationship of liposomal amphotericin B against *Candida auris* as a function of 48-hour change in log_10_ colony-forming units/mL from initial fungal load (horizontal dotted line) and maximum concentrations/minimum inhibitory concentrations. Abbreviations: CFU, colony-forming units; CLSI, Clinical and Laboratory Standards Institute; C_max_, peak concentration; L-AMB, liposomal amphotericin B; MIC, minimum inhibitory concentration; PK/PD, pharmacokinetic/pharmacodynamic.

After 48 hours of incubation, each drug-free control grew by >2.5 log_10_ CFU/mL, from 4.04 ± 0.24 log_10_ CFU/mL at t = 0 hours to 7.52 ± 0.56 log_10_ CFU/mL at t = 48 hours for all isolates. A ≥1.5–3 log_10_ CFU/mL reduction was obtained at C_max_ ≥8 mg/L for *C. auris* 60, 55, and 51 with low AMB/L-AMB MICs 0.5/0.125, 0.5/0.5, and 1/1 mg/L, respectively, whereas a 1 log_10_ CFU/mL reduction was found for the *C. auris* 52 with slightly higher AMB/L-AMB MIC and 2/4 mg/L at C_max_ ≥32 mg/L (Figure 4). When dilution of CFUs was taken into account and log_10_ CFU were analyzed, time-kill curves were shifted upward by approximately 1 log_10_ CFU due to a 20 times increase of the volume of the IC at 48 hours (data not shown). The in vitro exposure–effect relationship for *C. auris* isolates followed a sigmoid curve (R^2^ = 0.86). Mean C_max_/MIC associated with stasis was 9 (Figure 5). Similar PK/PD targets were found when log_10_ CFU was analyzed taking into account the dilution of CFUs.

#### Monte Carlo Simulation

**Figure 6.**
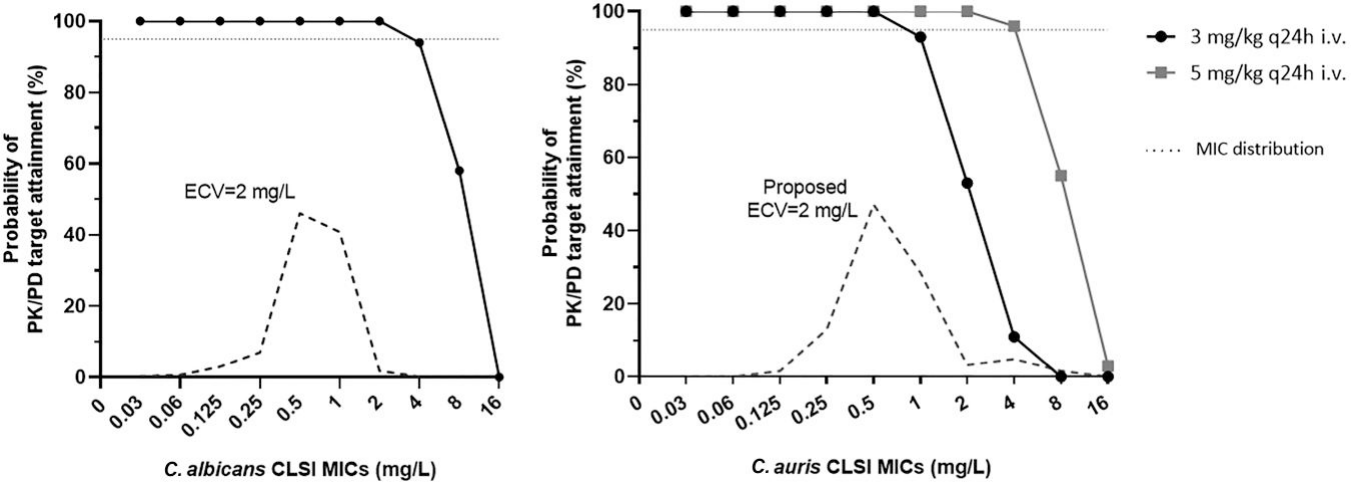
Probability of target attainment for 5000 patients receiving either the standard (3 mg/kg q24 intravenous [i.v.]) or higher (5 mg/kg q24 i.v.) liposomal amphotericin B dosage were simulated with Monte Carlo analysis for isolates with different Clinical and Laboratory Standards Institute amphotericin B minimum inhibitory concentrations. Horizontal line corresponds to 95% probability of target attainment. Abbreviations: CLSI, Clinical and Laboratory Standards Institute; ECV, epidemiological cutoff value; i.v., intravenous; MIC, minimum inhibitory concentration; PK/PD, pharmacokinetic/pharmacodynamic.

The PTA for the in vitro static PK/PD target with the standard L-AMB dose of 3 mg/kg q24h i.v. was high (>95%) for WT *C. albicans* with CLSI AMB MICs ≤2 mg/L (ECV 2 mg/L) and *C. auris* isolates with CLSI AMB MICs ≤1 mg/L not covering the presumed WT population of *C. auris* clinical isolates (proposed ECV 2 mg/L) [3], whereas with 5 mg/kg the PTA was >95% for *C. auris* CLSI AMB MIC ≤2 mg/L (Figure 6).

## DISCUSSION

An in vitro PK/PD dilution model was developed to explore the in vitro pharmacodynamics of L-AMB against *C. auris* isolates. Based on in vivo studies, it appears that the PK/PD index most closely related to AMB efficacy is C_max_/MIC, which also described well the L-AMB pharmacodynamics in our in vitro model [19]. The in vitro model was validated using the same *C. albicans* isolate previously used in an animal model of disseminated candidiasis, with the in vitro fungistatic PK/PD target of L-AMB (2.1 C_max_/MIC) being very close to the in vivo fungistatic PK/PD target in mouse kidneys (approximately 1.6–3.8 C_max_/MIC) taking into account L-AMB concentration at the site of infection; that is, the renal parenchyma (kidney area under curve [AUC] for stasis was 10 mg × hour/L for an isolate with MIC 0.25, ie, 40 AUC/MIC, which based on the 10.55–24.37 AUC/C_max_ ratio in kidney results in 1.6–3.8 C_max_/MIC) [10]. The PK/PD target of 2.1 C_max_/MIC resulted in high PTA for the entire WT population of *C. albicans* (ECV 2 mg/L) in line with clinical experience of good efficacy of L-AMB against *C. albicans* infections (90% success rate for *C. albicans* isolates) [20]. Other PK/PD targets previously found in animals, like the ∼1000 serum AUC/MIC (∼70 C_max_/MIC) corresponding to a static effect in mouse kidneys in neutropenic animal model [10], or the clinical 40 C_max_/MIC associated with partial response in children with candidemia [21] would result in low PTA for WT *C. albicans* isolates. Although AMB is fungicidal, a fungistatic target was the most clinically relevant as also found for echinocandins [22].

As L-AMB is minimally bound in serum and tissue homogenates, we were able to link the in vitro L-AMB concentrations with the in vivo concentrations at the site of infection and in particular mouse kidneys in a previously published animal model of disseminated candidiasis using the same *C. albicans* isolate [10]. Using serum drug exposures in mice, the PK/PD targets would be overestimated as L-AMB kidney concentrations are 10 times lower than serum concentrations in mice whereas in humans, high tissue concentrations were found in autopsy studies [23]. In addition, since most *Candida* infections are limited to the bloodstream, animal PK/PD targets should have been determined based on fungal burden in blood and not in kidneys for bloodstream infections. However, by linking drug concentrations with the fungal burden at the site of infection in animals, we were able to extrapolate to human cases of candidemia by simulating serum concentrations.

Consistent with other in vitro studies, L-AMB exhibited a concentration-dependent activity against *C. albicans* isolates. Specifically, increasing concentrations (≥2 times the MIC) led to a greater reduction in the fungal load (>2.5 log_10_ CFU/mL), with its fungicidal effect being fast, even 3 hours after drug addition [24]. Nevertheless, at lower L-AMB concentrations, regrowth was observed in vivo as early as 6 hours after treatment initiation, in agreement with the present study [10]. This regrowth may be due to shorter postantifungal effects observed at low AMB concentrations < MIC [25]. As for *C. auris* isolates, most studies focus on the activity of the conventional deoxycholate formulation [26], where AMB also showed weak but again concentration-dependent activity, with regrowth being observed early, even after 12 hours of treatment, as found in the present study. In vitro PK/PD data for conventional deoxycholate AMB formulation indicate that standard (0.7–1 mg/kg) doses were not effective against WT *C. auris* isolates with CLSI MICs 0.25–0.5 mg/L [27].

Regarding L-AMB and *C. auris*, a PK/PD target of 9 C_max_/MIC associated with stasis was found. Based on this target, high PTA (>95%) was found only for isolates with CLSI AMB MICs ≤1 mg/L with standard dose of 3 mg/kg q24h i.v., indicating that a higher dose is needed to cover the entire WT population of *C. auris* clinical isolates (proposed ECV 2 mg/L) [3]. The higher L-AMB dose of 5 mg/kg q24h i.v. was indeed associated with high PTA for *C. auris* isolates with CLSI AMB MIC ≤2 mg/L. To date, there are no established clinical breakpoints for AMB. However, the CDC has proposed a tentative breakpoint of ≥2 mg/L for resistance [9], which overlaps with the currently proposed ECV [3]. Regarding optimal doses, although there are no clinical data to support a higher dose, CDC recommends switching to L-AMB (5 mg/kg daily) if the patient is clinically unresponsive to echinocandin treatment or has persistent fungemia for >5 days [9]. Animal studies also demonstrated that a higher dose of 7.5 mg/kg L-AMB was effective (90% survival rates) in mice infected with a *C. auris* isolate with MIC 4 mg/L [28]. Thus, although the standard L-AMB dose of 3 mg/kg may be sufficient for isolates with MIC 1 mg/L, isolates with MIC 2 mg/L, which seem to be part of the WT population, will require the higher L-AMB dose of 5 mg/kg.

In conclusion, this study is the first PK/PD study exploring the pharmacodynamics of L-AMB against *C. auris* isolates. Based on our results, L-AMB was approximately 4 times less effective against *C. auris* compared to *C. albicans.* Monte Carlo simulations showed that a higher L-AMB dose of 5 mg/kg is needed to treat infections caused by *C. auris* isolates with CLSI MICs ≤2 mg/L, instead of the standard 3 mg/kg dose used for bloodstream infections by other *Candida* spp. Clinical studies are needed to evaluate the increased efficacy of 5 mg/kg L-AMB in comparison to the standard 3 mg/kg dosing regimen.

## Notes

***Acknowledgments***. We thank Drs Jacque Meis, Maiken Arendrup, and David Andes for kindly providing the isolates for this study.

***Financial support***. This study was sponsored by an unrestricted grant by Gilead Sciences Inc.
